# Structural characterization of human urea transporters UT-A and UT-B and their inhibition

**DOI:** 10.1126/sciadv.adg8229

**Published:** 2023-09-29

**Authors:** Gamma Chi, Larissa Dietz, Haiping Tang, Matthew Snee, Andreea Scacioc, Dong Wang, Gavin Mckinley, Shubhashish M. M. Mukhopadhyay, Ashley C. W. Pike, Rod Chalk, Nicola A. Burgess-Brown, Jean-Pierre Timmermans, Wouter van Putte, Carol V. Robinson, Katharina L. Dürr

**Affiliations:** ^1^ Structural Genomics Consortium, Nuffield Department of Medicine, University of Oxford, Roosevelt Drive, Oxford OX3 7DQ, UK.; ^2^ Centre for Medicines Discovery, Nuffield Department of Medicine, University of Oxford, Nuffield Department of Medicine Research Building, Oxford OX3 7FZ, UK.; ^3^ Department of Chemistry, University of Oxford, Oxford OX1 3TA, UK.; ^4^ Kavli Institute for Nanoscience Discovery, University of Oxford, Oxford OX1 3QU, UK.; ^5^ Laboratory of Cell Biology and Histology (CBH) at Antwerp Centre for Advanced Microscopy (ACAM), Department of Veterinary Sciences, University of Antwerp, Groenenborgerlaan 171, 2020 Antwerp, Belgium.; ^6^ PUXANO, Ottergemsesteenweg Zuid 713, 9000 Gent, Belgium.

## Abstract

In this study, we present the structures of human urea transporters UT-A and UT-B to characterize them at molecular level and to detail the mechanism of UT-B inhibition by its selective inhibitor, UTB_inh_-14. High-resolution structures of both transporters establish the structural basis for the inhibitor’s selectivity to UT-B, and the identification of multiple binding sites for the inhibitor will aid with the development of drug lead molecules targeting both transporters. Our study also discovers phospholipids associating with the urea transporters by combining structural observations, native MS, and lipidomics analysis. These insights improve our understanding of urea transporter function at a molecular level and provide a blueprint for a structure-guided design of therapeutics targeting these transporters.

## Teaser

Crystal and cryo-EM structures of human urea transporters UT-A and UT-B enable structure-based design of selective inhibitors.

## INTRODUCTION

Urea is a key molecule in the excretion of nitrogenous waste from complex organisms and plays a secondary role in a number of physiological processes, most notably the maintenance of osmotic balance at both cellular and broader physiological levels (*1*–*5*). Urea is primarily excreted in the kidney, where it passes the membrane filter in the glomerulus of the nephron then reabsorbed in the renal tubules. The second step of reabsorption is an important process in regulating the urine volume, hence blood pressure, as this facilitates reabsorption of water by osmotic gradient (*6*). While the initial excretion occurs through diffusion across the membrane, the subsequent reabsorption is mediated by urea transporters UT-A (*Hs*UT-A; SLC14A2) and UT-B (*Hs*UT-B; SLC14A1) which are differentially expressed in specific renal tubule compartments (*2*, *3*, *7*). This physiological role has made *Hs*UT-A and *Hs*UT-B targets for the development of a new class of salt-sparing diuretics, and a number of inhibitors selective to either member have been described (*8*–*15*). These inhibitors have been proven useful for not only the drug discovery programs but also uncovering other roles of urea transport in new disease areas. For example, cell volume dysregulation by *Hs*UT-B–facilitated urea transport was assessed with a selective inhibitor UTB_inh_-14 in neuroinflammation and in erythrocyte deformation (*4*, *16*, *17*), further adding to the profile of urea channel inhibitors as potential therapeutics for neurodegenerative diseases (*4*, *18*).

Crystal structures of bovine UT-B (*Bt*UT-B) and *Desulfovibrio vulgaris* urea transporter (*Dv*UT) have shown urea transporters to be homotrimers with a pore in each subunit (*19*, *20*). In these structures, 10 transmembrane (TM) helices of each subunit are arranged in a pseudo-C2-symmetry and allow urea transport in a channel-like mechanism. While these structures reveal the structural basis for substrate recognition and urea transport, information on other structural aspects of *Hs*UT-A and *Hs*UT-B, such as insights regarding interactions with small-molecule inhibitors and their selectivity, are not available.

In this study, we present high-resolution structures of human UT-A and UT-B determined with protein crystallography and cryo–electron microscopy (cryo-EM). While our structures show human urea transporters to have a similar overall architecture to *Bt*UT-B and *Dv*UT, we also observe substantial biophysical differences such as surface charges, with implications on their physiological roles. We also determined *Hs*UT-B in complex with UTB_inh_-14 inhibitor, which shows a different binding location and mode to those predicted in docking studies using *Bt*UT-B, and further characterized the biophysical properties of the inhibitor binding to *Hs*UT-B. Our structures also reveal key residue differences between *Hs*UT-A and *Hs*UT-B at the binding site of UTB_inh_-14, establishing the structural basis for inhibitor selectivity. Last, we present phospholipids closely associating with *Hs*UT-A and *Hs*UT-B with our cryo-EM structure and mass spectrometry (MS), revealing common features with structurally and functionally homologous ammonia transporters.

## RESULTS AND DISCUSSION

### Structures of human UT-A and UT-B reveal similar mechanism of urea transport

**Fig. 1.**
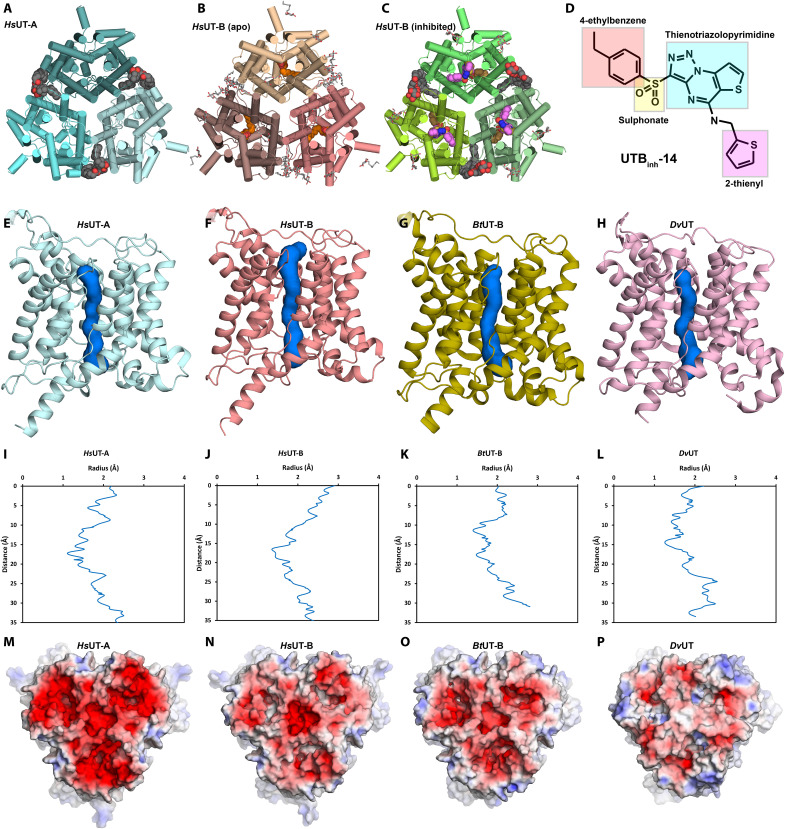
Overall structures of *Hs*UT-A and *Hs*UT-B are similar to each other and to their orthologs. (**A** to **C**) Overall structure models of *Hs*UT-A determined with cryo-EM (A), apo-*Hs*UT-B determined with crystallography (B), and inhibitor-bound *Hs*UT-B determined with cryo-EM (C). (**D**) Structure of UTBinh-14 used in this study. (**E** to **H**) Urea channel pores of *Hs*UT-A (E), *Hs*UT-B (F), *Bt*UT-B (G), and *Dv*UT (H). (**I** to **L**) Pore radius plots of *Hs*UT-A (I), *Hs*UT-B (J), *Bt*UT-B (K), and *Dv*UT (L). (**M** to **P**) Electrostatic surface representations (−5 kT/e in red to 5 kT/e in blue) of the extracellular side of *Hs*UT-A (M), *Hs*UT-B (N), *Bt*UT-B (O), and *Dv*UT (P).

We aimed to determine structures of both *Hs*UT-A and *Hs*UT-B to understand the functional differences between the two paralogs and to enable structure-guided design for selective inhibitors. Full-length *Hs*UT-B, while readily expressed in human embryonic kidney 293F cells (Thermo Fisher Scientific), failed to produce crystals with robust diffraction. Therefore, we engineered the construct for minimal truncation mimicking the limited proteolysis of *Bt*UT-B. Because a previous study has shown that glycosylation-knockout mutation N211I in *Hs*UT-B1 has no discernible effects on plasma membrane expression and urea uptake in *Xenopus* oocytes (*21*), we also removed N-linked glycosylation by site-directed mutagenesis. Together, these construct modifications yielded well-diffracting crystals, which resulted in a 2.4-Å x-ray structure (Fig. 1B). As the expression level for *Hs*UT-A was too low for crystallography, we opted for its structure determination by cryo-EM instead. The use of a graphene grid allowed us to overcome its inherent preferred orientation issues and low yield, eventually leading to an electrostatic potential (ESP) map of 2.9 Å nominal resolution (Fig. 1A and fig. S1D).

The overall structures of *Hs*UT-A and *Hs*UT-B are similar to each other and to *Bt*UT-B and *Dv*UT (*19*, *20*), showing the trimeric arrangement with 10 TM helices and two pore (P) helices per subunit (Fig. 1, A and B). *Hs*UT-A’s trimeric arrangement was an unexpected finding as our construct was equivalent to full-length UT-A1 isoform, which is a gene duplication of UT monomers (fig. S3). Closer observation of *Hs*UT-A’s map indicated that this sample was largely that of its N-terminal half (equivalent to *Hs*UT-A3) as most of its side chains matched with it (fig. S2, A, B, D, and E). However, of the 88 differing residues examined, there were a few notable exceptions such as A401/P863 where the map feature matched better to the C-terminal residues (fig. S2 F). Supporting the observation that both C- and N-terminal halves of *Hs*UT-A are present, MS of its tryptic digests showed the presence of both N-terminal and C-terminal peptides. Despite extensive data processing efforts, including static classification and particle heterogeneity analysis tools, it was not possible to separate the two species or to isolate maps with pseudo-heterooligomeric arrangement. As the majority of residues in the map are in line with the N-terminal half of *Hs*UT-A (i.e., *Hs*UT-A3), we built the model for its N-terminal sequences and based our structural analyses primarily on this model. For the 17 residues where the map was ambiguous or favoring C-terminal half, we left them without building side chains to make clear that the model may not properly represent the map for these regions.

In both structures, each subunit hosts a channel-like pore for urea transport and the channels are in the open state with approximately 2 to 3 Å in diameter at constriction formed by V69, T177, L292, and F342 (Fig. 1, I and J). This is similar to *Bt*UT-B and *Dv*UT, which also feature open pores of 2.5 and 2 Å at constriction (Fig. 1, K and L). This indicates that *Hs*UT-A3 and *Hs*UT-B in our structures are in similar urea-conducting states.

*Hs*UT-A3 shows significantly more negative surface charge on its extracellular side compared to other urea transporters (Fig. 1M). Whereas the extracellular surfaces of *Hs*UT-B and *Bt*UT-B are only mildly negatively charged and *Dv*UT close to net neutral (Fig. 1, N to P), nearly the entire extracellular surface of *Hs*UT-A shows electrostatic charges of less than −5 kT/e with Adaptive Poisson-Boltzmann Solver (APBS) electrostatics. *Hs*UT-A’s extracellular surface becomes less negatively charged with lower pH values, being similar to *Hs*UT-B’s when the pH is 5.0 (fig. S4). The urinary filtrate becomes significantly acidic in the inner medullary collecting duct (IMCD) where UT-A1 and UT-A3 are expressed with nominal pH as low as 5.5 (*22*, *23*), and local pH at the luminal surface is likely even lower as urine pH control is an active process (*24*). Hence, the surface charge of *Hs*UT-A3 in a physiological condition may vary depending on the IMCD luminal pH, which in turn is influenced by factors such as vasopressin. As negatively charged extracellular surface is a feature common to structurally related transporters of amines and urea (fig. S7), this surface charge may be an important feature of these proteins to attract their ligands.

### UTB_inh_-14 primarily inhibits UT-B by blocking its extracellular pore entry

UTB_inh_-14 is a highly selective inhibitor for UT-B, with over a thousand-fold selectivity over UT-A (*15*). Owing to such properties, it has been used as a chemical probe to study physiological roles of UT-B and is an attractive lead molecule for a drug discovery program (*4*). Therefore, our effort concentrated on obtaining the structure of *Hs*UT-B in complex with UTB_inh_-14 compound.

**Fig. 2.**
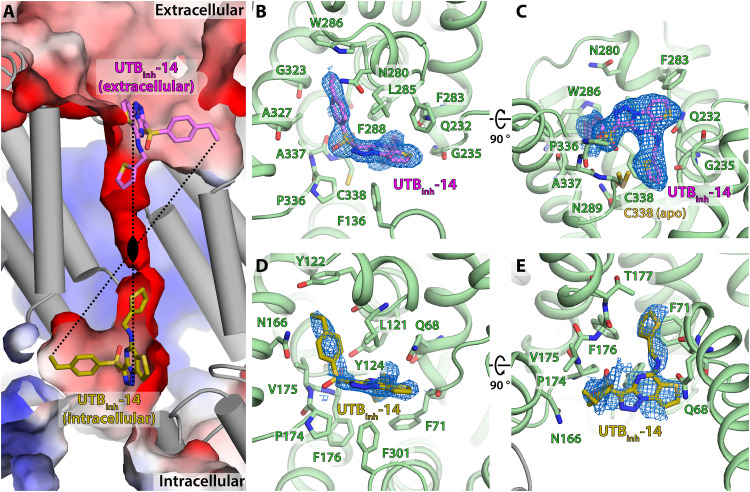
UTB_inh_-14 inhibits *Hs*UT-B at both extracellular and intracellular sides of its urea channel pore. (**A**) Transsectional view of *Hs*UT-B channel pore showing the locations of UTB_inh_-14. Black oval denotes the center of C2 pseudosymmetry axis of the inhibitor binding modes. (**B**) Extracellular view of the primary UTB_inh_-14 binding mode and (**C**) its transsectional view. (**D**) Intracellular view of the secondary UTB_inh_-14 binding mode and (**E**) its transsectional view.

We were not successful in determining the structure of *Hs*UT-B in the inhibitor-bound state with crystallography despite repeated attempts. Hypothesizing that this could be due to either the combination of low solubility and low affinity of UTB_inh_-14 or its low stability, we changed our structure determination technique to cryo-EM. This resulted in a refined ESP map with 2.6 Å nominal resolution, showing little conformational change (Fig. 1C) and revealing UTB_inh_-14 blocking the channel on the extracellular side (Fig. 2, B and C). Such a location is different from the prediction by docking studies, where it was thought to block UT-B from the intracellular side (*25*). In the crystal structure of apo-*Hs*UT-B, this site is occupied by a polyethylene glycol (PEG) molecule instead (fig. S5, A and B), so the high concentration of PEG400 in the crystallizing solution might have competed for this inhibitor-binding site and prevented us from obtaining UTB_inh_-14–bound structures with crystallography.

**Fig. 3.**
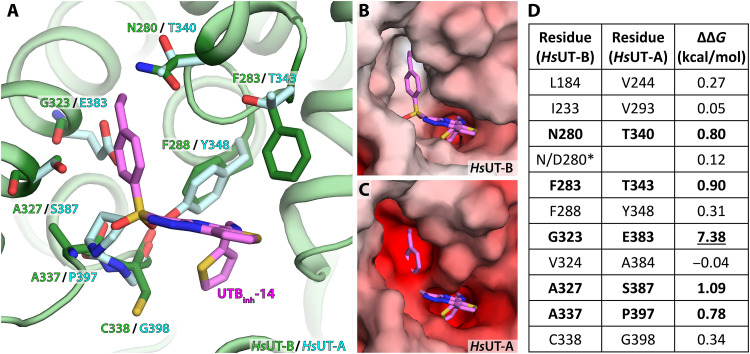
Structural basis for the selectivity of UTB_inh_-14 to UT-B. (**A**) Overlay of aligned *Hs*UT-A residues (cyan) against *Hs*UT-B residues (green) involved in UTB_inh_-14 binding. (**B**) Electrostatic surface charge representation of *Hs*UT-B at the UTB_inh_-14 binding pocket. (**C**) Electrostatic surface charge representation of *Hs*UT-A at the UTB_inh_-14 binding pocket. (**D**) Table of relative binding free-energy difference calculations for each residue mutation (from *Hs*UT-B to *Hs*UT-A).

UTB_inh_-14 fits well into this extracellular pocket, with its 2-thienyl group directly blocking the pore entry. The inhibitor forms hydrophilic interactions with the side chain of Q232 and the backbone carbonyl of A337 of *Hs*UT-B, and its 4-ethylbenzene group sits in a hydrophobic pocket formed by W286, F288, G323, and A327 (Fig. 2, B and C). Superimposing the *Hs*UT-A3 structure suggests that UTB_inh_-14 will not fit into its equivalent site due to its unfavorable environment (Fig. 3A): *Hs*UT-A3’s binding pocket is significantly more negatively charged than that of *Hs*UT-B, in particular for the 4-ethylbenzene binding region (Fig. 3, B and C). In silico binding free-energy calculations confirm this observation: Mutation of G323 in *Hs*UT-B (equivalent to E383 in *Hs*UT-A) to glutamic acid results in positive binding free-energy loss of 7.38 kcal/mol (from −14.32 to −6.94 kcal/mol), and A327S mutation results in loss of 1.09 kcal/mol (Fig. 3D). In addition, the 4-ethylbenzene group is in close proximity to N/D280 (~6 Å) (Fig. 2, B and C), whose variation is responsible for the Kidd blood group system (*26*). While mutating this residue from asparagine to aspartic acid does not change UTB_inh_-14’s binding free energy in our in silico calculations (0.12 kcal/mol), mutation to threonine (*Hs*UT-A equivalent) did lead to a minor loss of 0.8 kcal/mol, suggesting its involvement in the inhibitor binding. Therefore, it is possible for a hypothetical inhibitor based on UTB_inh_-14 to show selectivity toward certain Kidd blood group with consequences for its potential as a drug candidate.

Simple superimposition of *Hs*UT-A3 and inhibitor-bound *Hs*UT-B structure models suggests that the sulfonate ester group of the compound would sterically clash with *Hs*UT-A’s P397 (equivalent to A337 in *Hs*UT-B) (Fig. 3A). This sulfonate group at first appears to be important for the selectivity of UTB_inh_-14, as compounds featuring such moiety generally favor UT-B over UT-A (*15*, *27*), and even the one with highest UT-A affinity is only 13 times more potent for UT-A than for UT-B (*13*). However, in silico binding free-energy calculations suggest that this may not be a major influence on the compound selectivity, with an energy loss of only 0.78 kcal/mol (Fig. 3D). On closer examination, this is due to A337’s side chain facing away from the sulfonate group in *Hs*UT-B structure, hence mutation to proline making little difference to the compound binding (in contrast, side chain of *Hs*UT-A’s P397 faces toward the binding pocket). Therefore, while the sulfonate group may be a contributing factor in UTB_inh_-14’s selectivity, it may not be as significant as its 4-ethylbenzene group.

An interesting feature of the binding mode of UTB_inh_-14 is its 2-thienyl group at the channel pore entry (Fig. 2C). This group is in close proximity to C338 with its sulfur atom facing toward the residue, and this has forced the side chain of C338 to rotate away (with respect to the apo state) from the pore entry due to steric clash. If the side chain did not rotate away from the inhibitor, its thiol group would have been within 2.3 Å of the 2-thienyl group’s sulfur atom. Therefore, it is possible that the inhibitor could form a covalent bond with UT-B at C338 if the 2-thienyl group was replaced with a thiol group. Therefore, our structure of inhibitor-bound *Hs*UT-B suggests the possibility to generate irreversible covalent and selective inhibitors for UT-B.

### UTB_inh_-14 and β-OG can bind to UT-B on its intracellular side

Our cryo-EM map of UTB_inh_-14–bound *Hs*UT-B shows a small molecule feature on the intracellular side of the channel pore (Fig. 2, D and E). While its ESP feature is not as defined as that of UTB_inh_-14 on the extracellular side, it is still consistent with the shape of the inhibitor and not with any other potential molecules [e.g., β-dodecyl maltopyranoside (β-DDM)]. Real-space refinement with Phenix estimates its occupancy at 0.38, and the incomplete occupancy by UTB_inh_-14 suggests low binding affinity at this site compared to the extracellular one. While a published docking experiment predicted UTB_inh_-14 to also bind to the intracellular side (*25*), it is significantly different from our experimentally determined binding mode in that its 2-thienyl group is not directed toward the channel pore.

UTB_inh_-14’s binding mode on the intracellular side closely resembles that of the primary binding site on the extracellular side (Fig. 2A). Its 2-thienyl group is directed toward the channel pore, sterically blocking it, and its 4-ethylphenyl moiety is located in an adjacent hydrophobic pocket bounded by L116, Y122, and V175 (Fig. 2, D and E). These lead to UTB_inh_-14’s intracellular binding mode effectively being a rotational symmetry equivalent to its extracellular one, with the rotational axis located at the center of the pore (Fig. 2A). Such similarity of UTB_inh_-14’s extracellular and intracellular binding modes is not unexpected given that urea transporters feature a C2 pseudosymmetry around the same axis in the pore center. This also suggests that the binding of UTB_inh_-14 is driven by the sterics of the binding pocket (i.e., its shape) in addition to specific interactions between its functional groups and the protein.

While UTB_inh_-14 can bind to both intracellular and extracellular sides of UT-B, it is likely that its primary in vivo mode of inhibition is extracellular given our observations. First, drug molecules generally have higher bioavailability for extracellular surfaces than intracellular components. UTB_inh_-14’s apparent higher affinity (as inferred by higher occupancy) toward UT-B’s extracellular side would compound the bioavailability difference to saturate the extracellular binding pocket at far lower applied doses than it would the intracellular pocket in cell-based experiments. In addition, there is very little sequence difference between *Hs*UT-B and *Hs*UT-A in this intracellular pocket, and in silico mutation experiments of the few divergent residues (L116A and Y122H) showed less than 0.5 kcal/mol change in binding free energies (0.36 and 0.28 kcal/mol, respectively). Given that UTB_inh_-14 is a highly selective inhibitor for UT-B, such lack of difference in the intracellular pocket between UT-A and UT-B indicates that this is unlikely to be the main binding pocket for UTB_inh_-14 in a physiological setting.

Another ligand for the intracellular side of *Hs*UT-B in our structures is *n*-octyl-β-d-glucopyranoside (β-OG), which is present in the crystal structure of apo-*Hs*UT-B (fig. S5, C and D) since this short-chain detergent was used for crystallization experiments to improve crystal packing and diffraction but not for the EM studies. The glucoside group of β-OG forms hydrophilic interactions with several residues, including Q68, L364, and V367. On the other hand, its hydrophobic chain, which partially blocks the channel pore, is located in a mildly hydrophilic pocket formed by L121, Y122, N165, and V175. Such binding mode of β-OG closely matches that of UTB_inh_-14: β-OG’s hydrophilic glucoside positioned at the same site as the UTB_inh_-14’s thieno[2,3-e][1,2,3]triazolo[1,5-a]pyrimidine (TTP) group, which is also hydrophilic, and its hydrophobic tail at the same site as the UTB_inh_-14’s 4-ethylbenzene group (which is hydrophobic as well).

While the intracellular UTB_inh_-14 and β-OG in our *Hs*UT-B structures are likely artifacts of nonphysiological levels of each compound in a highly purified environment, they still provide useful clues about the potential binding poses of small molecules for de novo design of therapeutic inhibitors targeting this side of urea transporters. This can be particularly useful for UT-A inhibitor designs because (i) the extracellular side of UT-A1 is less accessible by drug molecules than the intracellular side in physiological settings since it faces the lumen of IMCD (*3*, *6*), and (ii) structural similarities between UT-A and UT-B allow extrapolation of UT-B’s interactions with UTB_inh_-14 and β-OG to UT-A inhibitor designs.

### Cryo-EM and MS identify phospholipids associating with UT-A and UT-B

**Fig. 4.**
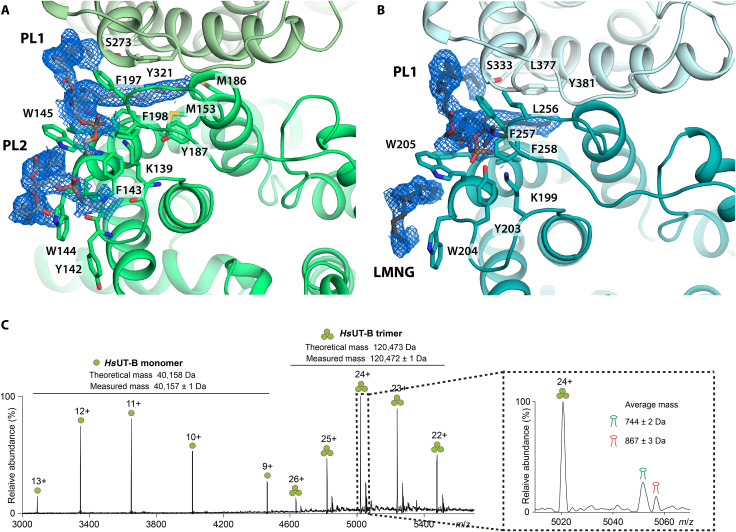
Cryo-EM structures of *Hs*UT-A and *Hs*UT-B show phospholipids occupying clefts at the subunit interfaces. (**A**) Extracellular view of phospholipids in inhibitor-bound *Hs*UT-B structure. PE lipids are modeled in. Light gray, phospholipids. (**B**) Extracellular view of phospholipids in *Hs*UT-A structure. Dark gray, phospholipid and LMNG at equivalent lipid site to *Hs*UT-B’s. (**C**) Native MS spectrum of *Hs*UT-B. Apo-*Hs*UT-B and two series of lipid-bound peaks are observed with average masses of 744 ± 2 Da and 867 ± 3 Da, respectively (insert).

Cryo-EM maps of both *Hs*UT-A and UTB_inh_-14–bound *Hs*UT-B structures show multiple features consistent with phospholipids. These features are located on the extracellular side of the TM surface, including one between subunits (Fig. 4, A and B). For each of the lipids at the subunit interface, one of the phospholipid tails extends far into a hydrophobic cleft formed at the interface of the subunits, bounded by L274 and Y321 of one subunit and M153, M186, and Y187 of the other subunit in the case of *Hs*UT-B (Fig. 4A). *Hs*UT-A and *Hs*UT-B appear to have similar lipid tail groups, with the lipid features in both *Hs*UT-A and *Hs*UT-B showing at least 16 carbon chains. For both proteins, the ends of the lipid tail groups are positioned close to the large internal cavities at the center of the trimers. Since the internal cavity of urea transporters is generally hypothesized to be filled with phospholipids (*28*), it is possible that this tail-binding cleft serves as a gate for lipids to move in and out of the cavity.

The map features for the phospholipid head groups are either very weak or nonexistent, especially for *Hs*UT-B’s (Fig. 4A), making their modeling inaccurate. Therefore, we could not determine the identity of the lipids from our structural data alone. We subsequently performed native MS analysis of *Hs*UT-B, where two adducts peaks were observed (Fig. 4C). In combination with lipidomics analysis, we identified these two adducts as phosphatidylethanolamines (PEs) and phosphatidylinositols (PIs) (fig. S6, A and B). Unlike PE, PI is only a minor component of mammalian plasma membrane, with the small amount rapidly phosphorylated to produce phosphatidylinositides for signaling, and even less is present on the outer side of the membrane bilayer (*29*, *30*). Therefore, the enrichment of PI suggests either specific binding to UT-B or its sequestration to cytoplasmic organelles such as the Golgi complex, which have significantly higher levels of PI (*31*).

The lipid binding modes in our structures are similar to that observed in a crystal structure of an *Escherichia coli* ammonia transporter *Ec*AmtB (fig. S6, C and D) (*32*), which is structurally and evolutionarily related to urea transporters. *Ec*AmtB’s annular lipids were identified as phosphatidylglycerols (PGs) and cardiolipin, whose head groups are net negatively charged like the PI lipids present in UT-B. Given the structural similarities, PIs may have a stabilizing influence on human urea transporters as PGs do on *Ec*AmtB, for example, by maintaining its homotrimeric state (*33*).

Of note, we do not observe lipid- or detergent-like feature in the central cavities of urea transporters despite hypotheses that they would be filled with lipids (*19*, *20*). Both crystal maps and cryo-EM reconstructions at subatomic resolutions of <4 Å commonly show “belts” of well-associating detergents and/or phospholipids around the TM surfaces of membrane proteins, such as those of *Ec*AmtB [Protein Data Bank (PDB) ID: 4NH2] (*34*), a lipid scramblase TMEM16F (6QP6) (*35*), a motor protein prestin (7LGU) (*36*), and a ligand-gated calcium channel TRPA1 (6PQQ) (*37*). Our urea transporter structures also show a number of lipid and detergent-like features around their TM surfaces, some of which are modeled; however, such features are lacking in their internal cavities, with only non–lipid-like disordered features seen at low contour levels. It also appears that these cavities would not be sterically favorable for phospholipids, as insufficient openings exist to accommodate the lipid head groups on either side of the bilayer for all urea transporters, including *Hs*UT-A and *Hs*UT-B. Nevertheless, it is still possible for them to be filled with lipids and/or have functional roles, as was the case with ATG9A whose central cavity is thought to aid in its lipid scramblase activity and in maintaining osmotic gradient across the membrane (*38*). Supporting this speculation is the structures of a functionally related (but structurally distinct) bacterial urea channel UreI in *Helicobacter pylori* (*39*, *40*), which feature lipid-like densities in their internal cavities. As this cavity is a consistent feature of urea transporters not present in their structural and functional homologs such as ammonia transporters *Ec*AmtB, *Hs*RhCG, and *Hp*UreI, as well as it will be interesting to study any physiological roles it may have.

In conclusion, both UT-A and UT-B are major targets for the development of a new class of diuretics, and their structural characterizations in our study will be valuable inputs for the drug discovery programs. We have identified the extracellular pocket as the primary binding site for the inhibitor UTB_inh_-14 with our structures, rationalized its selectivity to *Hs*UT-B over *Hs*UT-A, and provided specific ways to further improve the inhibitor design. In addition, our discovery of a second UTB_inh_-14 binding site on the intracellular side and the presence of β-OG in the apo-*Hs*UT-B structure can be used to design new inhibitors targeting this site, with potentials not only for *Hs*UT-B but also for *Hs*UT-A, which features nearly identical pocket.

Our human urea transporter structures and native MS also provide the basis for studying their close interactions with phospholipids. The identification of PI lipids associating with *Hs*UT-B is particularly interesting, and this suggests potential physiological roles such as protein stabilization, localization via lipid raft formation, or regulation by sequestration to PI-rich organelles. The last possibility is particularly compelling, since *Hs*UT-A1 and *Hs*UT-A3 are already known to be regulated in this way upon vasopressin-triggered phosphorylation (*41*, *42*). While we could not directly test such hypothesis with lipidomics MS on purified *Hs*UT-A due to its expression level issues in this study, this presents an exciting area of further investigation to understand the molecular physiology of urea transporters. The positioning of the lipid tails near the trimers’ central cavities is another interesting feature, and it remains to be investigated whether this cavity harbors lipids or has other physiological roles instead.

Last, our *Hs*UT-B structure allows mapping of exonic variants responsible for the wide range of Kidd blood group antigens (fig. S8). In addition to the major N/D280 variations for Jk(a) and Jk(b) blood types, there are a number of Jk(null) phenotypes caused by a number of point mutations (*26*, *43*, *44*). Mapping these variants on the *Hs*UT-B structure provides characterization of blood group antigens at molecular level, and this can also aid drug lead optimization programs by avoiding or using pockets near such variants. Our structures of human urea transporters therefore improve both the understanding of their function and the development of new diuretics targeting urea transport.

## MATERIALS AND METHODS

### Molecular biology, virus production, and protein expression

*Hs*UT-B with N-terminal truncation to aid crystallization and N211A mutation for deglycosylation (SLC14A1^Δ30N211A^) and full-length *Hs*UT-A were cloned into a pHTBV vector with C-terminal twin-Strep, 10-His, and green fluorescent protein tags. Baculoviruses for these constructs were generated following the standard protocol outlined in (*45*). *E. coli* DH10Bac cells were transformed with plasmids containing the target genes. Baculoviral DNA extracted from the cells was used to transfect Sf9 cells (Thermo Fisher Scientific) grown in Sf-900TM II media supplemented with 2% fetal bovine serum (Thermo Fisher Scientific) and incubated on an orbital shaker for 70 hours at 27°C. Produced baculovirus particles were harvested by centrifugation at 900*g* for 10 min and collecting the supernatants, and these were further amplified with Sf9 cells.

Each liter of Expi293F cell culture (Thermo Fisher Scientific) in Freestyle 293 Expression Medium (Thermo Fisher Scientific) was infected with 30 ml of P3 baculovirus-containing supernatant in the presence of 5 mM sodium butyrate. Cells were grown in an orbital shaker for 45 hours at 37°C and 8% CO_2_ before being harvested by centrifugation at 900*g* for 10 min, washed with phosphate-buffered saline, then centrifuged again. The cell-washed cell pellets were flash-frozen with liquid nitrogen (LN_2_), then stored at −80°C until needed.

### Protein purification

For the purification of apo-*Hs*UT-B, the following protocol was used. Whole-cell pellets expressing the target construct were resuspended to a total volume of 50 ml per 15 g of cell pellet with buffer A [20 mM Hepes (pH 7.5) and 150 mM NaCl] supplemented with 0.7% n-dodecyl beta-maltoside [(β-DDM) Generon] and 0.07% cholesteryl hemisuccinate (CHS; Generon). The cells were solubilized at 4°C for 1 hour with gentle rotation. Cell debris was pelleted by centrifugation at 45,000*g* for 1 hour. The clarified lysate was added to 0.5-ml bed volume of Strep-Tactin Superflow (IBA) per 100 ml of lysate and allowed to bind at 4°C for 1 hour. The resin was collected on a gravity-flow column and washed with buffer B (buffer A with 0.02% β-DDM and 0.002% CHS), then with buffer B supplemented with 2 mM adenosine 5′-triphosphate and 5 mM MgCl_2_. Protein was eluted with 10 CV of buffer B containing 5 mM d-desthiobiotin followed by tag cleavage by tobacco etch virus protease overnight and reverse purification. The samples were subjected to size exclusion chromatography with a Superose 6 Increase 10/300 column (GE Healthcare) pre-equilibrated with buffer C (buffer A with 0.7% β-OG). Peak fractions were pooled, concentrated to 100 μM and immediately used for crystallization experiments.

For the purification of UTB_inh_-14–bound *Hs*UT-B, all buffers were supplemented with 10 μM UTB_inh_-14 by adding 100 mM dimethyl sulfoxide stocks. Buffer B was used instead of buffer C for size exclusion chromatography, and the peak fractions were concentrated to 100 μM.

For the purification of *Hs*UT-A, same protocol as *Hs*UT-B was used, except 0.07% lauryl maltose neopentyl glycol (LMNG) replacing β-DDM at solubilization step, and 0.005% LMNG and 0.00025% CHS being used instead of other detergents in the rest of buffers. Peak fractions from size exclusion chromatography were concentrated to 1 μM for graphene-coated grid sample and 20 μM for Quantifoil grid sample.

### Crystallization of apo-*Hs*UT-B

Apo-*Hs*UT-B was crystallized by sitting-drop vapor diffusion method. Protein (1.9 mg/ml) was mixed using the Mosquito crystallization robot (TTP Labtech) with equal volume of precipitant containing 27% (v/v) PEG400, 0.1 M glycine (pH 9.5), and 0.05 M sodium chloride, in total volumes of 100, 150, or 200 nl. Successfully grown crystals were harvested using cryo-loops of appropriate size (Mitegen) and flash-frozen in liquid nitrogen. Diffraction data were collected at I24 Microfocus Beamline at Diamond Light Source (DLS, Harwell Science and Innovation Campus, Didcot, UK). Wavelength of x-ray radiation at the synchrotron was adjusted to 0.9686 Å. Raw x-ray diffraction data were analyzed using 3dii option of Xia2 (*46*). Details of the dataset collection parameters are available in Table 1.

**Table 1.** Data table for x-ray crystallography dataset of apo-*Hs*UT-B. RMSD, root mean square deviation.

Property	Apo-*Hs*UT-B
**Data collection and processing**	
Wavelength (Å)	0.9686
Resolution range (Å)	56.16–2.40 (2.46–2.40)
Space group	*P*6_3_
Unit cell (Å)	112.331 112.331 87.94 90 90 120
Total reflections	480,757 (28,936)
Unique reflections	24,811 (1834)
Multiplicity	19.4
Completeness (%)	100 (100)
Mean I/sigma(I)	1.49
Wilson B factor	50.8
*R*_merge_	0.217 (3.143)
*R*_meas_	0.229 (3.358)
CC_1/2_	0.997 (0.460)
**Refinement**	
Resolution range (Å)	47.33–2.40 (2.48–2.40)
*R*_work_	0.1754 (0.2826)
*R*_free_	0.2251 (0.3307)
Number of atoms	
All nonhydrogen atoms	2974
Macromolecules	2702
Ligands	201
Solvent	71
Number of protein residues	356
RMSD	
Bond lengths (Å)	0.009
Bond angles (°)	1.046
Ramachandran plot	
Favored (%)	97.46
Allowed (%)	2.54
Outliers (%)	0.00
Validation	
Molprobity score	1.22
Rotamer outliers (%)	0.00
Clash score	3.24
B factor	
Average (Å^2^)	61.0
Macromolecules (Å^2^)	59.27
Ligands (Å^2^)	92.03
Solvent (Å^2^)	58.71
Number of TLS groups	1
PDB ID	6QD5

### Model building and refinement of apo-*Hs*UT-B diffraction dataset

The structure was determined by molecular replacement with Phaser (*47*) using the *Bt*UT-B structure model (PDB ID: 4EZC) used as the search model. Nonconserved residues of the search model were cropped using Chainsaw (*48*) and the output was used as template for manual refinement in Coot (*49*). The models were subjected to cycles of manual refinements in Coot and Phenix real-space refine (*50*). Geometries of the models were verified with MolProbity function (*51*) in Phenix.

### Cryo-EM sample preparation and data collection

*Hs*UT-B at 100 μM was frozen on Quantifoil Au R1.2/1.3 300-mesh grids glow discharged on a high setting for 30 s, with plunge-freezing performed on a Vitrobot Mark IV (Thermo Fisher Scientific) set to 100% humidity and 4°C. *Hs*UT-A at 1 μM was frozen on Quantifoil Au R0.6/1 300-mesh grids with a graphene monolayer support using a protocol similar to Han *et al.* (*52*) and Naydenova *et al.* (*53*). Graphene grids were glow discharged on a low setting for 5 s, with plunge-freezing performed on Vitrobot Mark IV set to 100% humidity and 4°C. *Hs*UT-A at 10 μM was frozen on Quantifoil Au R1.2/1.3 300-mesh grids glow discharged on a high setting for 30 s, with pluge-freezing performed on a Vitrobot Mark IV set to 100% humidity and 4°C.

The cryo-EM datasets were collected on a Titan Krios (Thermo Fisher Scientific) operating at 300 keV at Oxford Particle Imaging Centre (Oxford, UK), where dose-fractionated micrographs were collected on a K2 detector with beam tilt collection mode on Serial EM. Details for each collected dataset are available in Table 2.

**Table 2.** Data table for cryo-EM data collection, refinement, and validation statistics of UTB_inh_-14–bound *Hs*UT-B and apo-*Hs*UT-A.

	*Hs*UT-B (inhibited)	*Hs*UT-A (graphene coated)	*Hs*UT-A (normal/tilt)
**Data collection**			
Microscope	Titan Krios	Titan Krios	Titan Krios
Detector	K2	K2	K2
Voltage (kV)	300	300	300
Magnification	105,000	105,000	105,000
Collection mode	Counting	Counting	Counting
Electron exposure (e/Å^2^)	39.6	52.6	53.3/52.0
Number of frames	40	50	50
Pixel size	0.82	0.82	0.82
Defocus range (μm; steps)	−0.8 to −2.6 (−0.2)	−1.0 to −2.6 (−0.2)	−1.0 to −2.6 (−0.2)/−1.6 to −1.8 (0.2)
Number of movies	8221	4431	4237/7028
**Data processing**			
Initial number of particles	2,288,882	723,119	1,581,566/2,527,272
Number of particles after 2D classification	284,410	180,511	137,735/81,266
Symmetry	C3	C3	C3
Number of particles used for 3D refinement	258,739	180,448	218,993
Map resolution [Å; Fourier Shell Correction (FSC) threshold = 0.143]	2.6	2.9	3.0
Resolution range (Å)	2.4–22.1	2.6–5.6	2.7–32.3
Map sharpening B factor (Å^2^)	109	138	141
**Refinement**			
Model resolution(Å; FSC threshold = 0.5)	2.7	3.1	
**Model composition**			
Nonhydrogen atoms	9378	8487	
Protein residues	1065	1005	
Ligands	66	24	
RMSD			
Bond lengths (Å)	0.012	0.009	
Bond angles (°)	1.156	0.852	
Validation			
Molprobity score	1.72	1.64	
Clash score	7.08	7.97	
Rotamer outliers	0.35	0.98	
Ramachandran plot			
Favored (%)	95.18	96.70	
Allowed (%)	4.82	2.97	
Disallowed (%)	0.00	0.33	
			
EMDB code	EMD-16112	EMD-16110	EMD-16111
PDB code	8BLP	8BLO	

### Cryo-EM data processing

Micrographs were imported to Cryosparc 2.14 and motion-corrected with its Patch Motion Correction function (*54*). After defocus estimation with its Patch CTF function, particles were picked initially with blob picking and extracted with 100 pixel box size. After iterative two-dimensional (2D) classifications, classes with rare particle orientations were selected, which were then subjected to Topaz training and extraction jobs (*55*). These particles were then cleaned up with duplicated particle removal function, followed by extraction to 100 pixel box size and 2D classifications. The polished particles were used for ab initio model reconstruction function on Cryosparc with two classes generated for further polishing. These were then used for two cycles of nonuniform refinement, per-particle motion correction, and CTF refinement, then the reconstructions with highest nominal resolutions on Fourier Shell Correlation curve and map quality on visual inspection were used for further analyses.

### Model building and refinement of cryo-EM datasets

The *Hs*UT-B structure model from crystallography was fitted to the ESP map of the UTBinh-14–bound *Hs*UT-B. In the case of the *Hs*UT-A dataset, the *Hs*UT-B structure model’s sequence was replaced with *Hs*UT-A’s using the Chainsaw function in CCP4i2 (*48*, *56*), which was then fitted to its ESP map. The models were subjected to cycles of manual refinements in Coot and Phenix real-space refine (*49*, *50*). Geometries of the models were verified with MolProbity function in Phenix (*51*). Model coordinates and restraints for the UTBinh-14 compound were generated using AceDRG (*57*). UTB_inh_-14 models were fitted to ESP features consistent with the compound, with its occupancy estimated with Phenix.

### Relative binding free energy calculations

The model of UTB_inh_-14–bound *Hs*UT-B structure was loaded to ICM-Pro (Molsoft) and converted to an ICM-compliant model with pH set to 7.5. Relative binding energy differences (ΔΔ*G*) of residue mutation from *Hs*UT-B to equivalent amino acids in *Hs*UT-A were calculated with Try Mutations function of ICM-Pro (*58*).

### Native MS

*Hs*UT-B samples were buffer exchanged to 200 mM ammonium acetate with 2× CMC β-OG, loaded into a gold-coated needle, and introduced into a Q-Exactive UHMR mass spectrometer (Thermo Fisher Scientific). The following parameters were used: Capillary voltage was set to 1.2 kV, source fragmentation was set to 25 eV, injection flatapole was set to 5 V, inter flatapole was set to 4 V, bent flatapole was set to 2 V, resolution was set to 17500 at mass/charge ratio (*m*/*z*) 200, higher-energy C-trap dissociation (HCD) energy was set to 100 eV, and trapping gas pressure was set to 7.5.

### Lipidomics analysis

The bound lipids of *Hs*UT-B samples were extracted by chloroform-methanol (2:1, v/v) as described previously (*59*) and dried using a speedVac vacuum concentrator (Thermo Fisher Scientific). The samples was then dissolved in 80% methanol. For liquid chromatography–tandem MS (LC-MS/MS) analysis, lipids were separated on a C18 column (Acclaim PepMap 100, C18, 75 μm × 150 mm, 3 μm, Thermo Fisher Scientific) by a Dionex UltiMate 3000 RSLC Nano System connected to an Orbitrap Eclipse Tribrid mass spectrometer (Thermo Fisher Scientific). The mobile phase A is acetonitrile:H_2_O (60:40), 10 mM ammonium formate, and 0.1% formic acid, and mobile phase B is isopropanol:acetonitrile (90:10), 10 mM ammonium formate, and 0.1% formic acid. Lipids were separated with a gradient of 32 to 99% buffer B at a flow rate of 300 nl/min. The Orbitrap Eclipse was operated in negative mode. Spray voltage was set to −2.1 kV and ion transfer tube temperature was at 320°C. For data-dependent acquisition, full MS scan range was set to 200 to 2000 with a resolution of 120,000. Fragments were acquired using a normalized collision energy at 25/30/35. Phospholipid identifications were performed manually using Xcalibur 4.4.

### In-gel digest MS

The identity of proteins in solution or in gel bands excised following SDS–polyacrylamide gel electrophoresis (PAGE) was confirmed by tryptic digestion and tandem MS (LC-MS/MS). SDS-PAGE gel bands were excised as 1 mm by 4 mm slices using a gel cutting tip (GeneCatcher, web Scientific) and stored in 10% MeOH at 4°C. Before digestion, the methanol solution was removed and replaced with 100% acetonitrile for 2 min. The solution was then removed and replaced with 100 μl of 100 mM NH_4_HCO_3_ (pH 8.0). One microliter of 1 M dithiothreitol was added and incubated at 56°C for 40 min. Four microliters of 1 M iodoacetamide was then added and the reaction incubated at ambient temperature in the dark for 20 min. A further 1 μl of 1 M dithiothrietol, 200 μl of 100 mM NH_4_HCO_3_, and 1 μl of trypsin solution (sequencing grade, Sigma-Aldrich; 1 mg/ml in 0.01 M HCl) was then added. Tryptic digestion proceeded at 37°C for 16 hours and was terminated by addition of 3 μl of formic acid. LC-MS/MS was performed using a Dionex U3000 Nano HPLC coupled to a Bruker Amazon ETD ion trap mass spectrometer. One microliter of tryptic digest was loaded on to a 200 μm inside diameter × 5 cm PS-DVB monolith column (PepSwift, Dionex Corp.) A linear gradient of 0% B to 15% B was developed over 5 min, followed by a second linear gradient from 15% B to 40% B over 2 min. The column was washed at 90% for 2 min and then equilibrated at 90% B for a further 6 min. Solvent A was 2% (v/v) acetonitrile, 0.1% formic acid in water, and solvent B was 80% acetonitrile and 0.1% formic acid. The flow rate was 2.5 μl/min. The mass spectrometer was operated in positive ion, standard enhanced mode with a scan rate of 8100 *m*/*z* per sec and a scan range of 250 to 1800 *m*/*z*. The trap accumulation time was 200 ms and the accumulation target was 200,000 counts. Data-dependent peptide fragmentation was performed in Auto MSMS mode. Compound extraction and peptide deconvolution was performed using the DA data analysis program (Bruker Daltonik). Database searching was performed using the Mascot 2.2.04 search algorithm (Matrix Science) with the following search parameters: Charge states +2, +3; MS tolerance 1.5 Da; MSMS tolerance 0.5 Da; UniProt_SwissProt database without taxonomic restrictions.

## Supplementary Materials

### This PDF file includes:

Figs. S1 to S9 Supplementary Text References
