# Supplementary material for: Structural characterization of human urea transporters UT-A and UT-B and their inhibition
Source: Sci Adv. 2023 Sep 29;9(39):eadg8229. doi: 10.1126/sciadv.adg8229 (PMC10541013; doi:10.1126/sciadv.adg8229)
Supplement: Supplementary file 1 — Figs. S1 to S9 Supplementary Text References [file sciadv.adg8229_sm.pdf]

Science Advances
AAAS

Supplementary Materials for

Structural characterization of human urea transporters UT-A and UT-B and their inhibition

Gamma Chi *et al.*

Corresponding author: Gamma Chi, gamma.chi@cmd.ox.ac.uk

Sci. Adv. **9**, eadg8229 (2023)
DOI: 10.1126/sciadv.adg8229

This PDF file includes:

Figs. S1 to S9
Supplementary Text
References

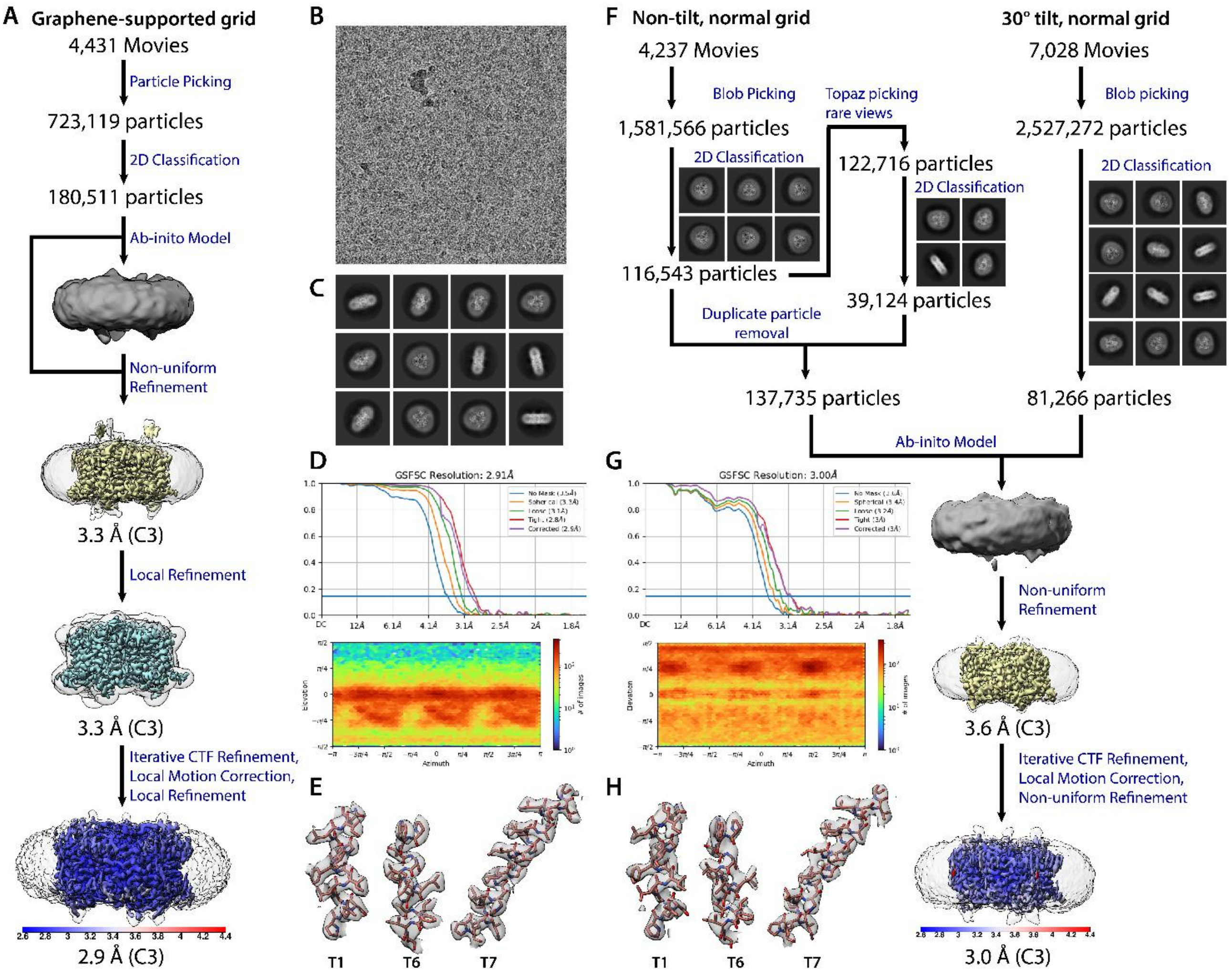

Supplementary Figure S1. Flowchart of the processing workflow for the *Hs*UT-A cryo-EM datasets. A) Processing workflow of *Hs*UT-A dataset collected on a graphene-supported EM grid. B) Representative raw micrograph of *Hs*UT-A on a graphene-supported EM grid**. C)** 2D classes of *Hs*UT-A on graphene-supported grid. **D)** Fourier shell correlation (FSC) curve graph and angular distribution map of the dataset with graphene support. **E)** Electrostatic potential (ESP) maps and models of peripheral transmembrane helices T1, T6 and T7 showing the quality of map and model fit. **F)** Processing workflow of *Hs*UT-A datasets collected on a normal grid, without tilt (left) and with 30° tilt (right). **G)** FSC curve graph and angular distribution map of the tilt/non-tilt dataset. **H)** ESP maps and models of T1, T6 and T7 for the tilt/non-tilt dataset. Map anisotropy with vertical streaks and poorer model fits can be seen despite near-identical nominal resolution to graphene dataset.

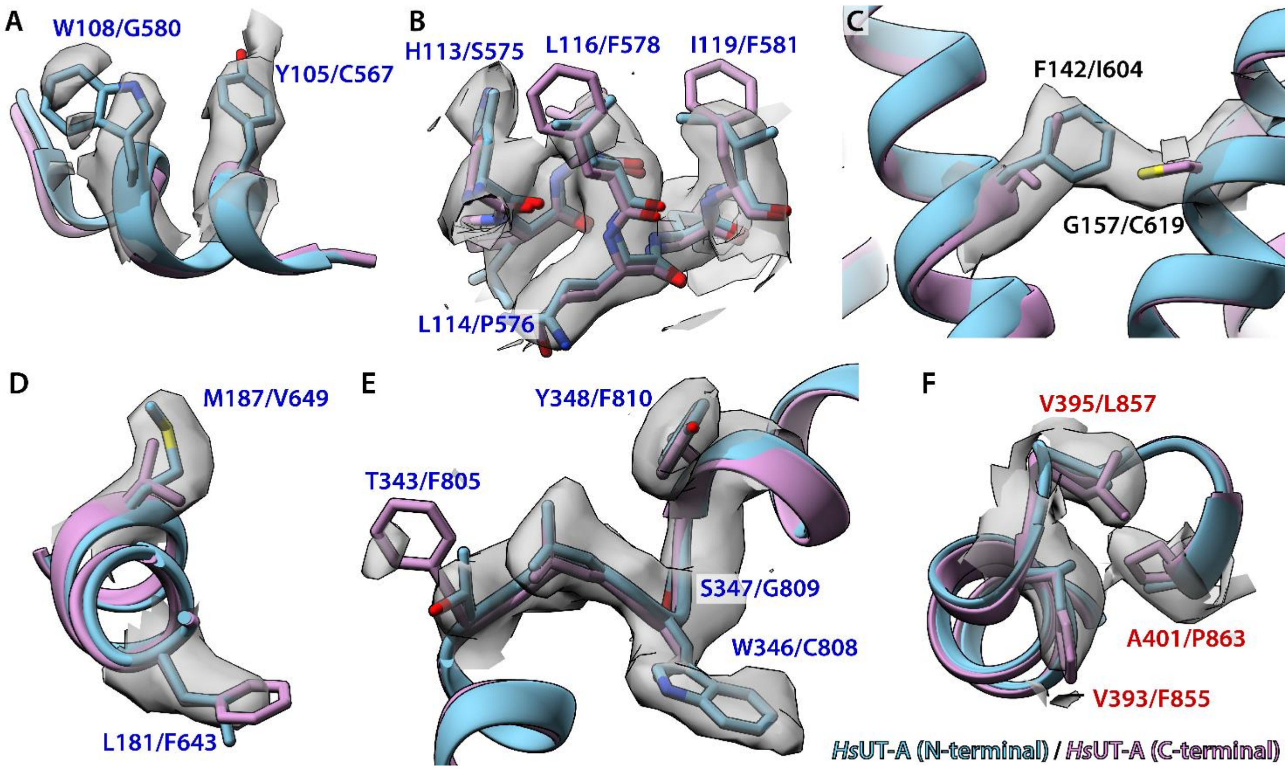

Supplementary Figure S2. Comparison of *Hs*UT-A models for its N-terminal (cyan) and C-terminal (pink) domains fitted to *Hs*UT-A cryo-EM map. Each section shows residues where the domains' sequences are different: **A)** W108/C567 (N/C domains) and W108/G580; **B)** H113 to I119/S575 to F581; **C)** F142/I604 and G157/C619; **D)** L181/F643 and M187/V649; **E)** T343 to Y348/F805 to F810; and **F)** V393 to A401/F855 to P863. Blue - residues where the cryo-EM map favours N-terminal domain model; Black – residues where the map is ambiguous (i.e. either model can fit); Red – residues where the map favours C-terminal domain model.

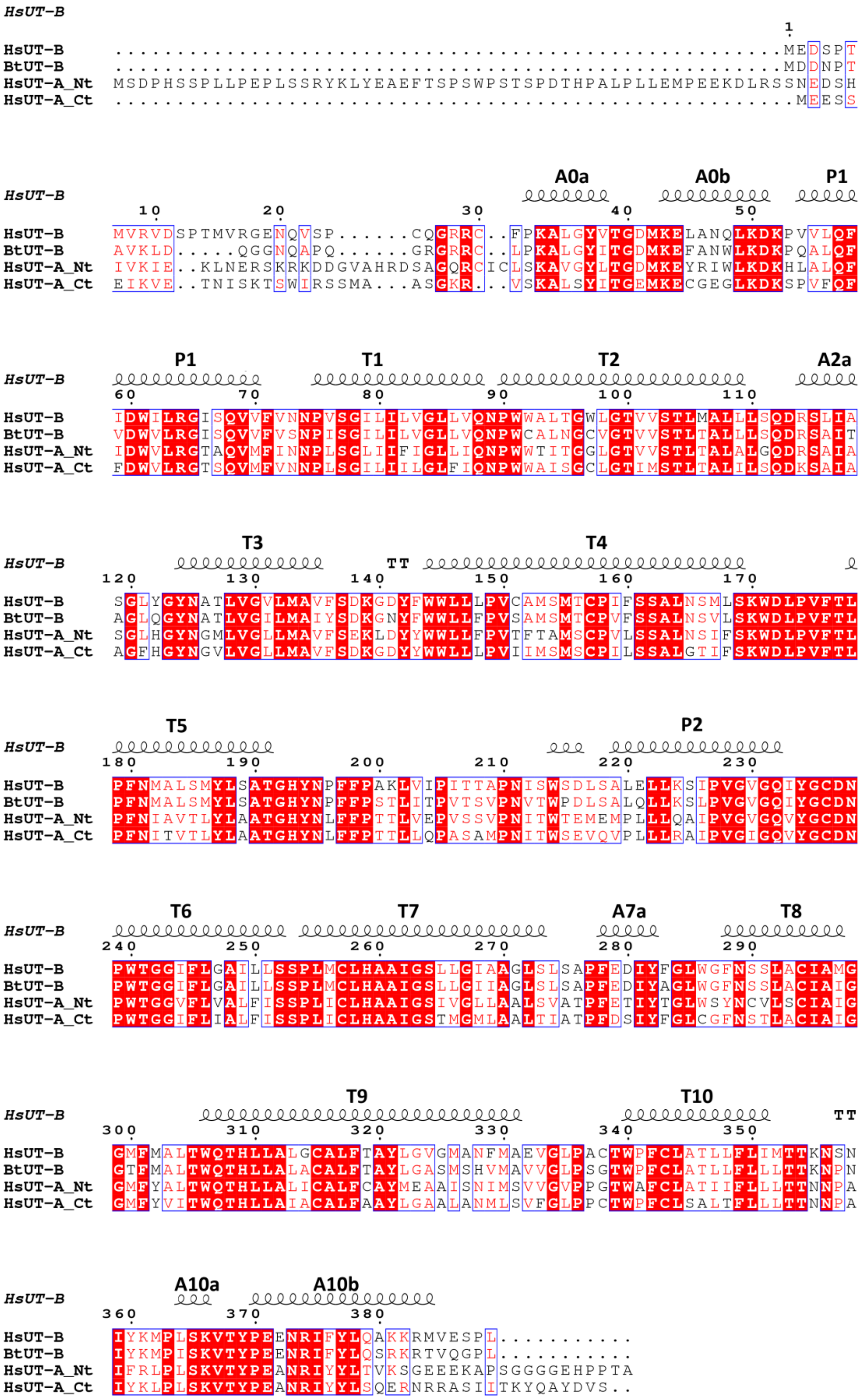

Supplementary Figure S3. Sequence alignment of *Hs*UT-B, *Bt*UT-B, N-terminal domain of *Hs*UT-A and C-terminal domain of *Hs*UT-A. *Hs*UT-A's N-terminal domain is equivalent to *Hs*UT-A3 isoform, and its C-terminal domain is equivalent to *Hs*UT-A2 isoform.

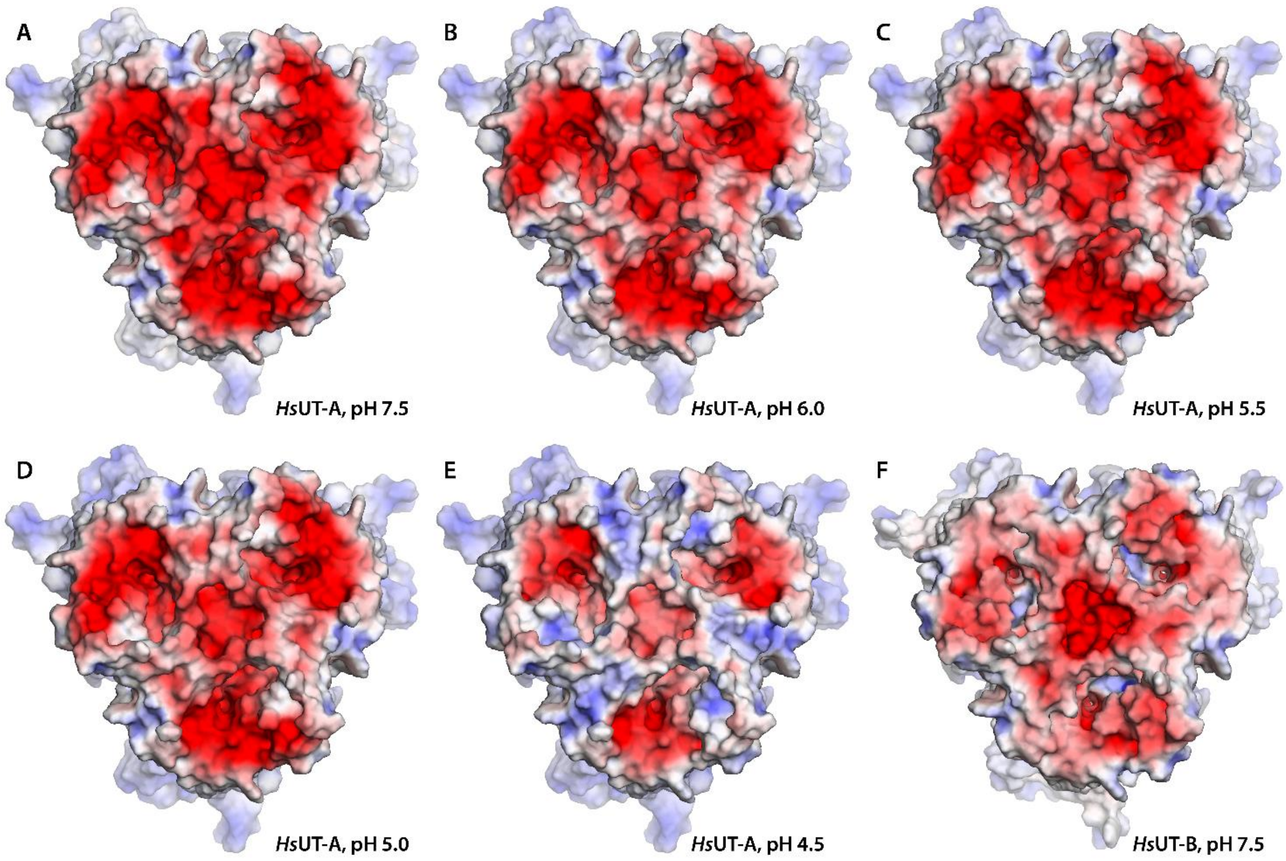

Supplementary Figure S4. Electrostatic surface charge representations of *Hs*UT-A at various pH levels (extracellular view). A) pH 7.5, **B)** pH 6.0, **C)** pH 5.5, **D)** pH 5.0 and **E)** pH 4.5. **F)** Electrostatic surface charge representation of *Hs*UT-B at pH 7.5 for comparison. (-5 kT/e in red to 5 kT/e in blue).

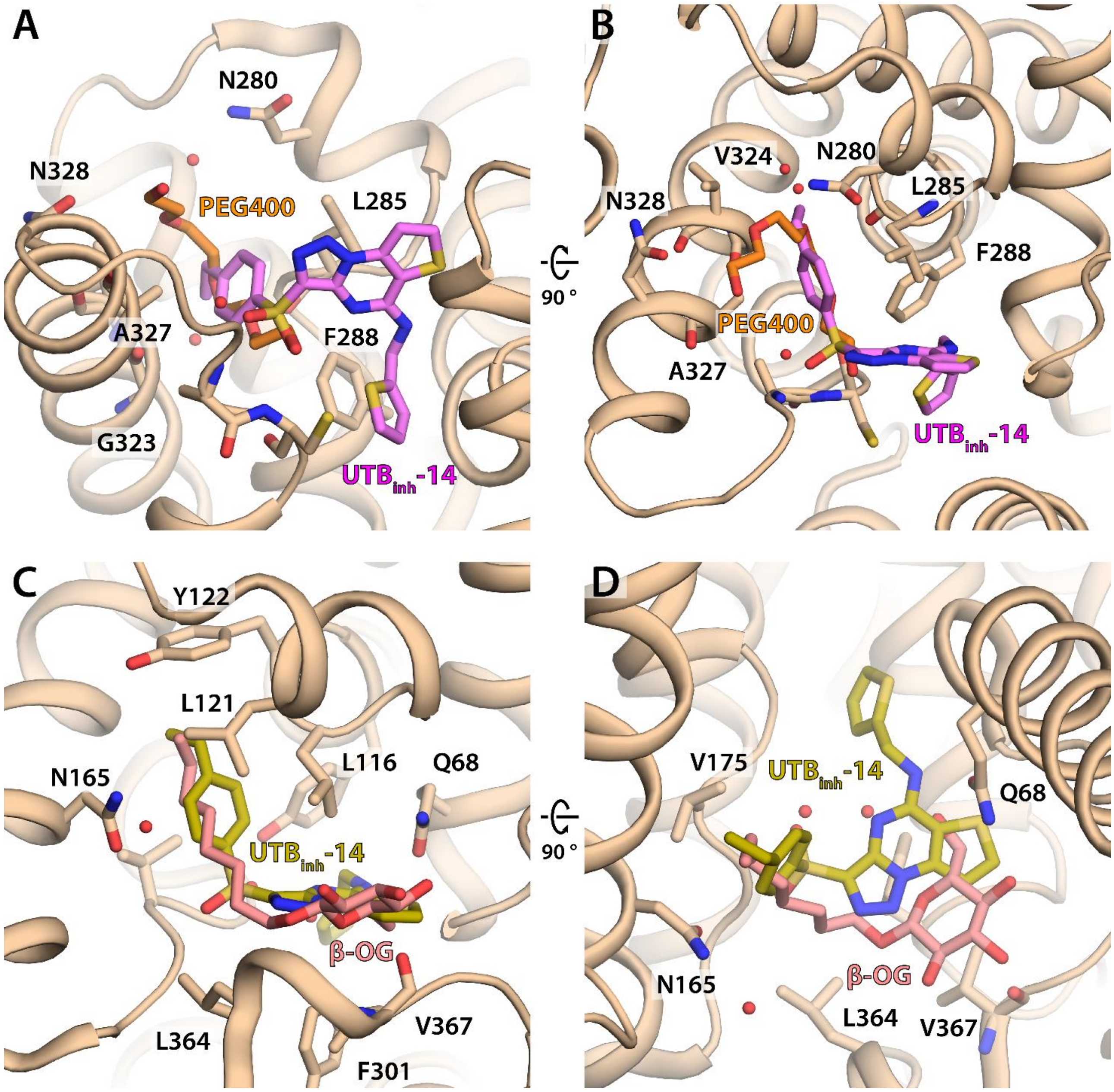

Supplementary Figure S5. PEG400 and β-OG molecules occupying extracellular and cytoplasmic UTB$_{inh}$-14-binding pockets in the crystal structure of apo-*Hs*UT-B.

A, B) Extracellular (**A**) and transsectional (**B**) views of the extracellular binding pocket. UTB$_{inh}$-14 (purple) from the cryo-EM structure is shown for reference. **C, D)** Cytoplasmic (C) and transsectional (D) views of the cytoplasmic binding pocket. UTB$_{inh}$-14 (olive) from the cryo-EM structure is shown for reference.

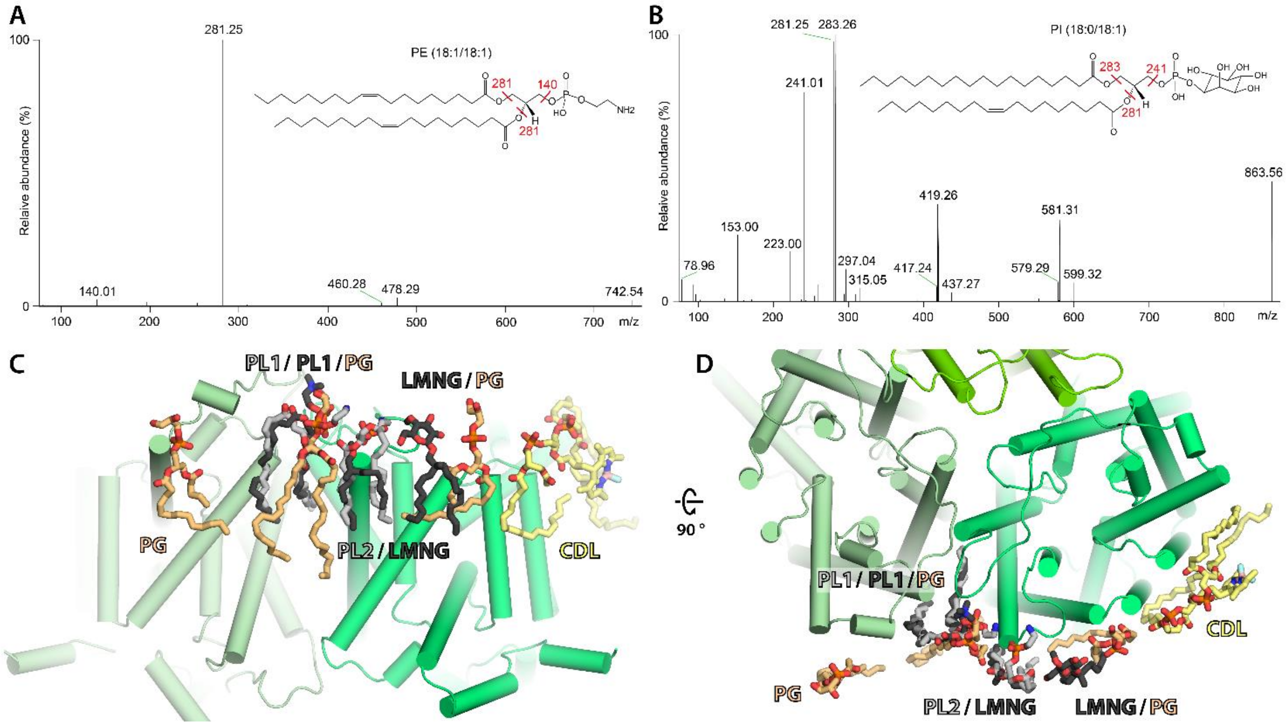

Supplementary Figure S6. Lipidomics analysis of *Hs*UT-B and comparison of lipid binding sites in *Hs*UT-B, *Hs*UT-A and AmtB. A, B) Lipidomics analysis of the lipid extractions from *Hs*UT-B sample and identification of PE and PI. **A)** the MS/MS identification of PE (18:1/18:1), **B)** the MS/MS identification of PI (18:0/18:1). **C, D)** Transsectional (C) and extracellular (D) views of *Hs*UT-B with phospholipid models from *Hs*UT-A and *Ec*AmtB superimposed. Light grey – phospholipids in *Hs*UT-B structure; Dark grey – phospholipids (PL) and detergent LMNG in *Hs*UT-A structure; Light orange – phosphatidylglycerols (PG) in AmtB (PDB ID: 4NH2); Light yellow – cardiolipin (CDL) in AmtB (PDB ID: 6B21)

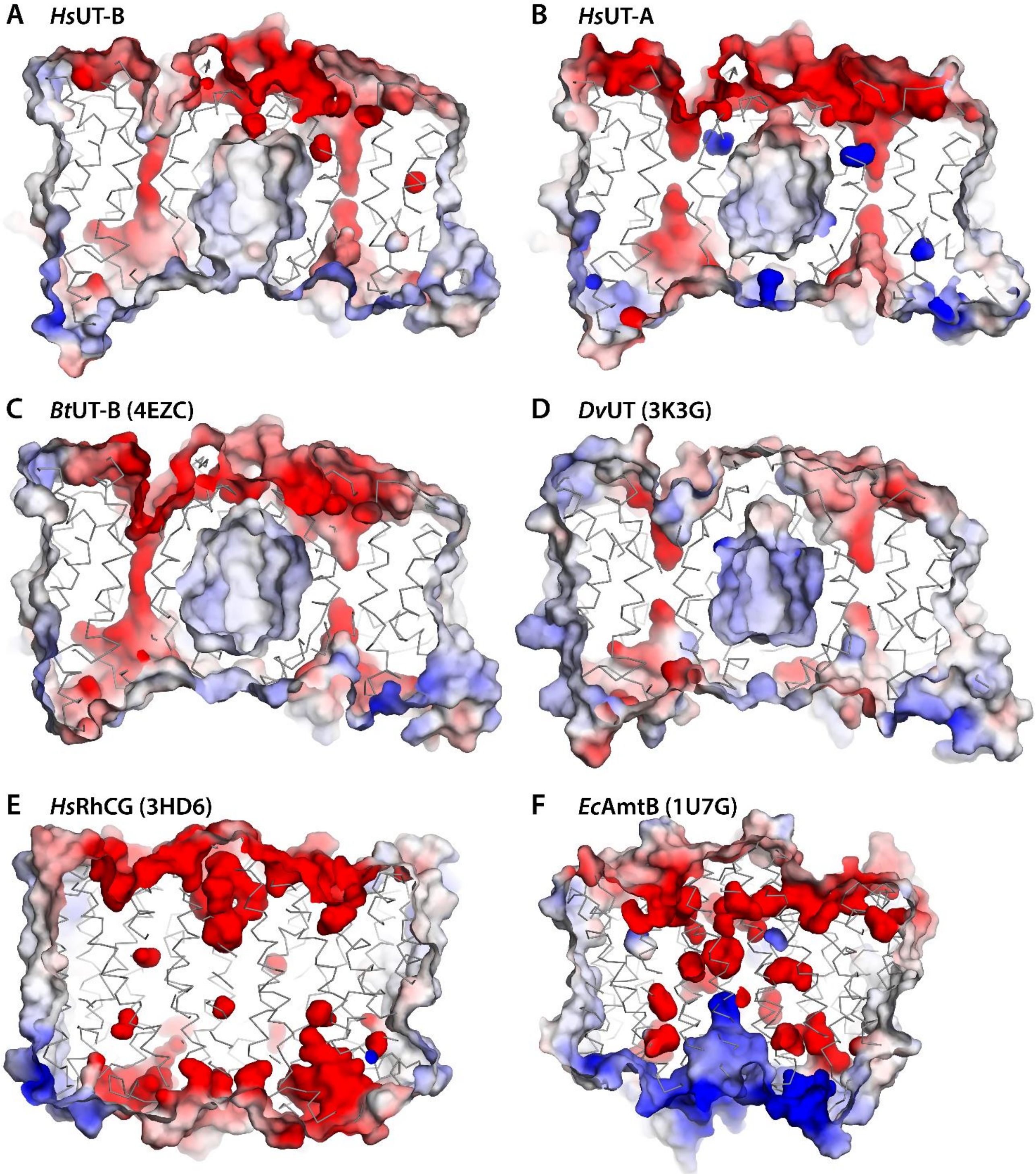

Supplementary Figure S7. Transsectional views of the electrostatic surface charge representations of various amine/amide transporters showing their channel pores and internal cavities.

A) *Hs*UT-B, **B)** *Hs*UT-A, **C)** *Bt*UT-B (PDB ID: 4EZC), **D)** *Dv*UT (3K3G), **E)** *Hs*RhCG (3HD6), and **F)** *Ec*AmtB 1U7G). Urea transporters commonly feature large internal cavities with a small or no opening on either side of the membrane (A – D), whereas ammonia transporters do not (E, F). The channel pore is also more continuous for urea transporters (A – D) than for ammonia transporters (E, F). (-5 kT/e in red to 5 kT/e in blue).

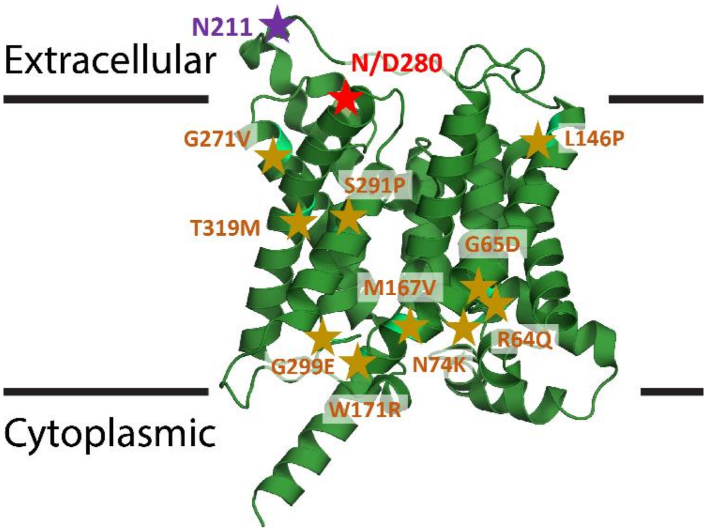

Supplementary Figure S8. Positions of *Hs*UT-B antigenic sequence variations.
Purple – glycosylation site at N211 involved in ABO blood type. Red – N/D280 variations responsible for Jk(a/b) blood type. Gold – Sequence variations responsible for Jk(null) blood types.

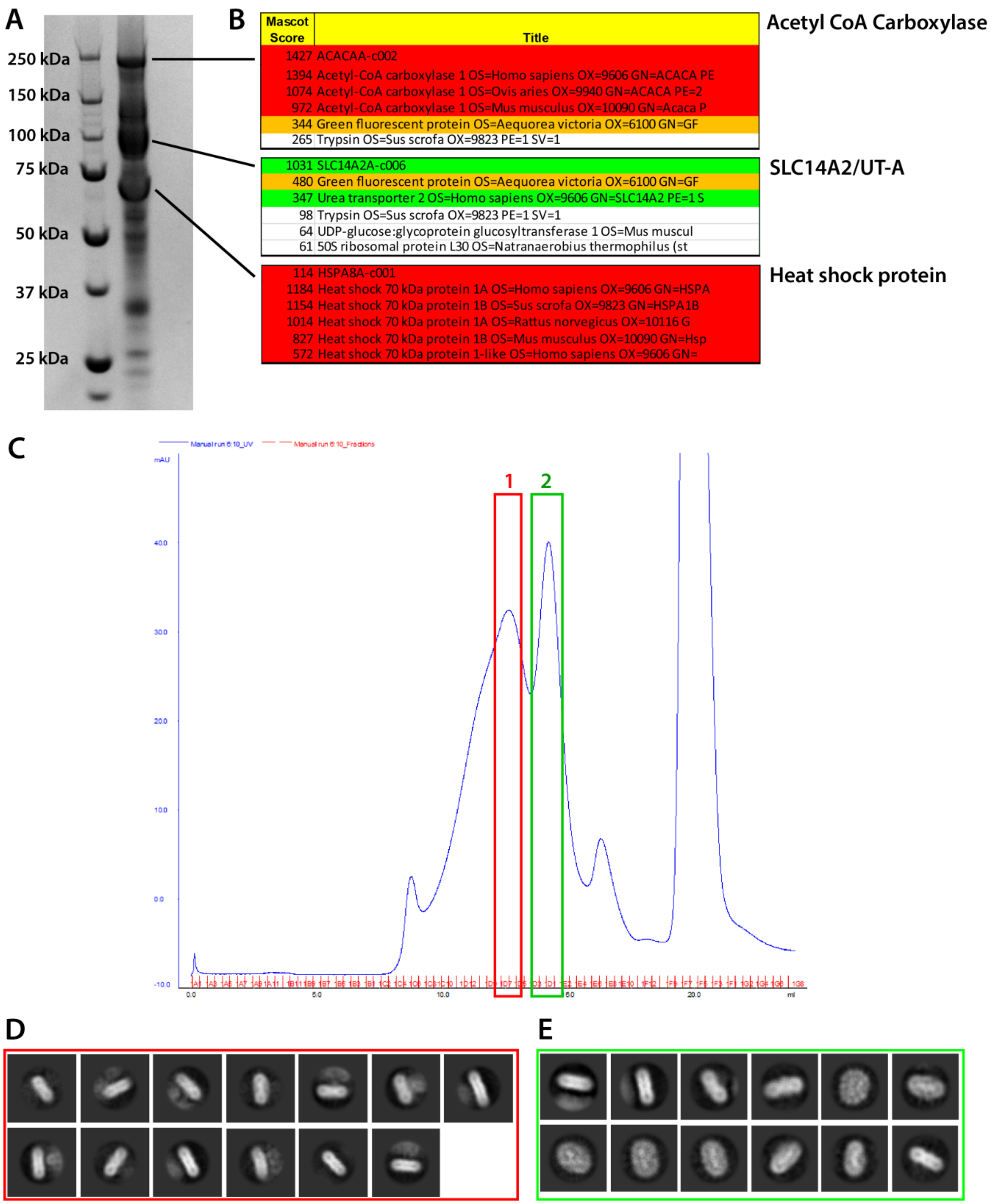

Supplementary Figure 9. Detailed analysis of UT-A purification. **A)** UT-A after Strep tag purification shows three major bands, at 250 kDa, 100 kDa and 70 kDa. **B)** Gel band mass spectrometry of the SDS-PAGE gel shows that the 250 kDa band is acetyl-CoA carboxylase, 100 kDa band is UT-A, and 70 kDa band is HSP8. **C)** Size exclusion chromatography of purified UT-A. Red - first peak eluting at 13 mL. Green – second peak eluting at 14 mL. **D)** 2D classification result of first peak from SEC. **E)** 2D classification result of second peak from SEC. Particle sizes are nearly identical between the first and second peak fractions.

Supplementary Results and Discussion

UTB$_{inh}$-14 has a destabilising effect on human UT-B *in vitro.*

*Hs*UT-B shows little conformational change upon UTB$_{inh}$-14 binding and its affinity appears significantly lower with purified protein compared to UT-B in cells as neither co-crystallisation nor crystal soaking resulted in the inhibitor-bound structure. Therefore, we sought to characterise the biophysical effect of UTB$_{inh}$-14 on *Hs*UT-B's stability with a combination of thermal shift assays and native mass spectrometry. A thermal shift assay based on tryptophan fluorescence showed relative destabilisation of *Hs*UT-B in 100 µM UTB$_{inh}$-14 by 1.5℃ compared to DMSO-only control. A similar result was obtained for FSEC-based thermal shift assay as well, where ~1.0℃ reduction in thermostability was observed with inhibitor-added *Hs*UT-B. Interestingly, with the latter method, a smaller species of *Hs*UT-B, likely representing its monomeric state, was observed in higher proportions at the protein transition temperature. This was subsequently confirmed by native mass spectrometry of *Hs*UT-B subsequently confirmed this, which found higher ratio of monomers to trimers for the inhibitor-bound state compared to the apo state (fig. S6). These suggest that UTB$_{inh}$-14 destabilises *Hs*UT-B by disrupting its oligomeric state. This observation cannot be easily explained by the inhibitor-bound structure since both binding sites for UTB$_{inh}$-14 are distant from the oligomer interface, and there is little structural difference between apo and inhibitor-bound states of *Hs*UT-B. In addition, the native MS experiment did not detect mass differences between apo and UTB$_{inh}$-14-bound *Hs*UT-B (fig. S6). This is probably due to the removal of the inhibitor during the detergent-stripping process, and suggests low affinity with high off-rate for the UTB$_{inh}$-14 compound. These results establish UTB$_{inh}$-14 as a weak inhibitor for *Hs*UT-B with a destabilising effect on the protein *in vitro* in contrast to the cell-based assay data in the literature, where its potency was described in the nanomolar range.

Such discrepancy in compound affinity measurements between cell-based assays and those with purified proteins is not uncommon. In this case, the cause could be attributed to a combination of the low solubility of UTB$_{inh}$-14 and an artificial environment where the nominal concentrations for both protein and inhibitor are at non-physiological levels.

Graphene grid strategy improves cryo-EM dataset angular distribution.

*Hs*UT-A in an LMNG/CHS detergent environment has a severe orientation bias with the "flat" side facing the air/water interface on standard Quantifoil 300-mesh grids with 1.2/1.3 hole spacing. As 2D classification of collected dataset showed promising results in terms of particle homogeneity and stability, we decided to address the orientation distribution issue using two approaches (fig. S1). In the first approach, we collected two datasets for *Hs*UT-A in the standard Quantifoil grids, 4,237 movies without tilt and 7,028 movies with 30° tilt, but with all other parameters set identically (fig. S1 F – H). Then we processed the datasets separately at first, then combined them to assess whether this would reduce the map anisotropy problem. The non-tilt dataset led to a reconstruction with a nominal resolution of 3.6 Å upon refinement, however significant anisotropy was present, whereas the map from the 30° tilt dataset reached only 4.3 Å but showed somewhat reduced anisotropy. Combining particles from the two datasets improved both the resolution (3.0 Å) and the particles' angular distribution (fig. S1 G), however there was still some level of anisotropy present when the map was visually inspected (fig. S1 H). We hypothesise that this is due to the degradation of dataset quality caused by the high angle tilt, which would lead to the loss of high-resolution information in most particles sourced from this dataset. This observation has a similar precedence with haemagglutinin datasets (*1*), where reduction in high resolution information was observed with increasing tilt. The map anisotropy was poor enough to prevent accurate modelling of residues in some regions, so a new approach to the preferred orientation problem was required.

In our second approach, we applied the same batch of *Hs*UT-A sample on a Quantifoil grid coated with a continuous graphene monolayer followed by collection of 4,431 movies with identical parameters as the previous collections (fig. S1 A – E). Processing this dataset led to a 2.9 Å reconstruction of *Hs*UT-A, and while a low level of preferred orientation was present, it was not serious enough to affect the map quality (fig. S1 D). Visual inspection also confirms this, with no anisotropy present in the map (fig. S1 E). As combining all three datasets did not lead to any improvement in the quality of the reconstruction, the *Hs*UT-A model was built and refined based on the map from the graphene dataset.

REFERENCES AND NOTES

1. T. Titko, L. Perekhoda, I. Drapak, Y. Tsapko, Modern trends in diuretics development. *Eur. J. Med. Chem.* **208**, 112855 (2020).

2. G. Stewart, The emerging physiological roles of the SLC14A family of urea transporters. *Br. J. Pharmacol.* **164**, 1780–1792 (2011).

3. C. P. Smith, Mammalian urea transporters. *Exp. Physiol.* **94**, 180–185 (2009).

4. A. C. Jones, F. Pinki, G. S. Stewart, D. A. Costello, Inhibition of urea transporter (UT)-B modulates LPS-induced inflammatory responses in BV2 microglia and N2a neuroblastoma cells. *Neurochem. Res.* **46**, 1322–1329 (2021).

5. S. Azouzi, M. Gueroult, P. Ripoche, S. Genetet, Y. Colin Aronovicz, C. le van Kim, C. Etchebest, I. Mouro-Chanteloup, Energetic and molecular water permeation mechanisms of the human red blood cell urea transporter B. *PLOS ONE* **8**, e82338 (2013).

6. J. D. Klein, M. A. Blount, J. M. Sands, Urea transport in the kidney. *Compr. Physiol.* **1**, 699–729 (2011).

7. J. M. Sands, M. A. Blount, Genes and proteins of urea transporters. *Subcell. Biochem.* **73**, 45–63 (2014).

8. S. A. Amin, S. Nandi, S. K. Kashaw, T. Jha, S. Gayen, A critical analysis of urea transporter B inhibitors: Molecular fingerprints, pharmacophore features for the development of next-generation diuretics. *Mol. Divers.* **26**, 2549–2559 (2022).

9. S. Wang, Y. Xu, Y. Zhao, S. Zhang, M. Li, X. Li, J. He, H. Zhou, Z. Ge, R. Li, B. Yang, N-(4-acetamidophenyl)-5-acetylfuran-2-carboxamide as a novel orally available diuretic that targets urea transporters with improved PD and PK properties. *Eur. J. Med. Chem.* **226**, 113859 (2021).

10. S. Nandi, S. Sanyal, S. A. Amin, S. K. Kashaw, T. Jha, S. Gayen, Urea transporter and its specific and nonspecific inhibitors: State of the art and pharmacological perspective. *Eur. J. Pharmacol.* **911**, 174508 (2021).

11. M. Li, Y. Zhao, S. Zhang, Y. Xu, S. Y. Wang, B. W. Li, J. H. Ran, R. T. Li, B. X. Yang, A thienopyridine, CB-20, exerts diuretic activity by inhibiting urea transporters. *Acta Pharmacol. Sin.* **41**, 65–72 (2020).

12. Y. Zhao, M. Li, B. Li, S. Zhang, A. Su, Y. Xing, Z. Ge, R. Li, B. Yang, Discovery and optimization of thienopyridine derivatives as novel urea transporter inhibitors. *Eur. J. Med. Chem.* **172**, 131–142 (2019).

13. S. Lee, O. Cil, E. Diez-Cecilia, M. O. Anderson, A. S. Verkman, Nanomolar-potency 1,2,4-triazoloquinoxaline inhibitors of the kidney urea transporter UT-A1. *J. Med. Chem.* **61**, 3209–3217 (2018).

14. F. Li, T. Lei, J. Zhu, W. Wang, Y. Sun, J. Chen, Z. Dong, H. Zhou, B. Yang, A novel small-molecule thienoquinolin urea transporter inhibitor acts as a potential diuretic. *Kidney Int.* **83**, 1076–1086 (2013).

15. M. O. Anderson, J. Zhang, Y. Liu, C. Yao, P. W. Phuan, A. S. Verkman, Nanomolar potency and metabolically stable inhibitors of kidney urea transporter UT-B. *J. Med. Chem.* **55**, 5942–5950 (2012).

16. B. Huang, H. Wang, D. Zhong, J. Meng, M. Li, B. Yang, J. Ran, Expression of urea transporter b in normal and injured brain. *Front. Neuroanat.* **15**, 591726 (2021).

17. X. Geng, T. Lei, H. Zhou, W. Yao, W. Xin, B. Yang, The knockout of urea transporter-B improves the hemorheological properties of erythrocyte. *Clin. Hemorheol. Microcirc.* **65**, 249–257 (2017).

18. R. R. Handley, S. J. Reid, R. Brauning, P. Maclean, E. R. Mears, I. Fourie, S. Patassini, G. J. S. Cooper, S. R. Rudiger, C. McLaughlan, P. J. Verma, J. F. Gusella, M. MacDonald, H. J. Waldvogel, C. S. Bawden, R. L. M. Faull, R. G. Snell, Brain urea increase is an early Huntington's disease pathogenic event observed in a prodromal transgenic sheep model and HD cases. *Proc. Natl. Acad. Sci. U.S.A.* **114**, E11293-E11302 (2017).

19. E. J. Levin, Y. Cao, G. Enkavi, M. Quick, Y. Pan, E. Tajkhorshid, M. Zhou, Structure and permeation mechanism of a mammalian urea transporter. *Proc. Natl. Acad. Sci. U.S.A.* **109**, 11194–11199 (2012).

20. E. J. Levin, M. Quick, M. Zhou, Crystal structure of a bacterial homologue of the kidney urea transporter. *Nature* **462**, 757–761 (2009).

21. N. Lucien, F. Sidoux-Walter, N. Roudier, P. Ripoche, M. Huet, M. M. Trinh-Trang-Tan, J. P. Cartron, P. Bailly, Antigenic and functional properties of the human red blood cell urea transporter hUT-B1*. *J. Biol. Chem.* **277**, 34101–34108 (2002).

22. K. Ishibashi, S. Sasaki, N. Yoshiyama, T. Shiigai, J. Takeuchi, Generation of pH gradient across the rabbit collecting duct segments perfused in vitro. *Kidney Int.* **31**, 930–936 (1987).

23. H. H. Bengele, J. H. Schwartz, E. R. McNamara, E. A. Alexander, Chronic metabolic acidosis augments acidification along the inner medullary collecting duct. *Am. J. Physiol.* **250**, F690–694 (1986).

24. R. C. Stanton, D. C. Boxer, J. L. Seifter, Expression of Na(+)-H+ exchange and ATP-dependent proton extrusion in growing rat IMCD cells. *Am. J. Phys.* **258**, C416–C420 (1990).

25. A. S. Verkman, C. Esteva-Font, O. Cil, M. O. Anderson, F. Li, M. Li, T. Lei, H. Ren, B. Yang, Small-molecule inhibitors of urea transporters. *Subcell. Biochem.* **73**, 165–177 (2014).

26. S. Lawicki, R. B. Covin, A. A. Powers, The Kidd (JK) blood group system. *Transfus. Med. Rev.* **31**, 165–172 (2017).

27. M. H. Levin, R. de la Fuente, A. S. Verkman, Urearetics: A small molecule screen yields nanomolar potency inhibitors of urea transporter UT-B. *FASEB J.* **21**, 551–563 (2007).

28. E. J. Levin, M. Zhou, Structure of urea transporters. *Subcell. Biochem.* **73**, 65–78 (2014).

29. J. P. Zewe, A. M. Miller, S. Sangappa, R. C. Wills, B. D. Goulden, G. R. V. Hammond, Probing the subcellular distribution of phosphatidylinositol reveals a surprising lack at the plasma membrane. *J. Cell Biol.* **219**, e201906127 (2020).

30. J. G. Pemberton, Y. J. Kim, J. Humpolickova, A. Eisenreichova, N. Sengupta, D. J. Toth, E. Boura, T. Balla, Defining the subcellular distribution and metabolic channeling of phosphatidylinositol. *J. Cell Biol.* **219**, e201906130 (2020).

31. D. Casares, P. V. Escribá, C. A. Rosselló, Membrane lipid composition: Effect on membrane and organelle structure, function and compartmentalization and therapeutic avenues. *Int. J. Mol. Sci.* **20**, 2167 (2019).

32. A. Laganowsky, E. Reading, T. M. Allison, M. B. Ulmschneider, M. T. Degiacomi, A. J. Baldwin, C. V. Robinson, Membrane proteins bind lipids selectively to modulate their structure and function. *Nature* **510**, 172–175 (2014).

33. K. Gupta, J. A. C. Donlan, J. T. S. Hopper, P. Uzdavinys, M. Landreh, W. B. Struwe, D. Drew, A. J. Baldwin, P. J. Stansfeld, C. V. Robinson, The role of interfacial lipids in stabilizing membrane protein oligomers. *Nature* **541**, 421–424 (2017).

34. S. Khademi, J. O'Connell III, J. Remis, Y. Robles-Colmenares, L. J. W. Miercke, R. M. Stroud, Mechanism of ammonia transport by Amt/MEP/Rh: Structure of AmtB at 1.35 Å. *Science* **305**, 1587–1594 (2004).

35. C. Alvadia, N. K. Lim, V. Clerico Mosina, G. T. Oostergetel, R. Dutzler, C. Paulino, Cryo-EM structures and functional characterization of the murine lipid scramblase TMEM16F. *eLife* **8**, e44365 (2019).

36. J. Ge, J. Elferich, S. Dehghani-Ghahnaviyeh, Z. Zhao, M. Meadows, H. von Gersdorff, E. Tajkhorshid, E. Gouaux, Molecular mechanism of prestin electromotive signal amplification. *Cell* **184**, 4669–4679.e13 (2021).

37. Y. Suo, Z. Wang, L. Zubcevic, A. L. Hsu, Q. He, M. J. Borgnia, R.R. Ji, S.Y. Lee, Structural insights into electrophile irritant sensing by the human TRPA1 channel. *Neuron* **105**, 882–894.e5 (2020).

38. S. Maeda, H. Yamamoto, L. N. Kinch, C. M. Garza, S. Takahashi, C. Otomo, N. V. Grishin, S. Forli, N. Mizushima, T. Otomo, Structure, lipid scrambling activity and role in autophagosome formation of ATG9A. *Nat. Struct. Mol. Biol.* **27**, 1194–1201 (2020).

39. Y. Cui, K. Zhou, D. Strugatsky, Y. Wen, G. Sachs, Z. H. Zhou, K. Munson, pH-dependent gating mechanism of the *Helicobacter pyloriurea* channel revealed by cryo-EM. *Sci. Adv.* **5**, eaav8423 (2019).

40. D. Strugatsky, R. McNulty, K. Munson, C.K. Chen, S. M. Soltis, G. Sachs, H. Luecke, Structure of the proton-gated urea channel from the gastric pathogen *Helicobacter pylori*. *Nature* **493**, 255–258 (2013).

41. M. A. Blount, J. D. Klein, C. F. Martin, D. Tchapyjnikov, J. M. Sands, Forskolin stimulates phosphorylation and membrane accumulation of UT-A3. *Am. J. Physiol. Renal Physiol.* **293**, F1308–F1313 (2007).

42. C. Zhang, J. M. Sands, J. D. Klein, Vasopressin rapidly increases phosphorylation of UT-A1 urea transporter in rat IMCDs through PKA. *Am. J. Physiol. Renal Physiol.* **282**, F85–F90 (2002).

43. J. R. Hamilton, Kidd blood group system: A review. *Immunohematology* **31**, 29–35 (2015).

44. T. Onodera, K. Sasaki, H. Tsuneyama, K. Isa, K. Ogasawara, M. Satake, K. Tadokoro, M. Uchikawa, JK null alleles identified from Japanese individuals with Jk(a−b−) phenotype. *Vox Sang.* **106**, 382–384 (2014).

45. P. Mahajan et al., in *Structural Genomics: General Applications*, Y. W. Chen, C.-P. B. Yiu, Eds. (Springer US, 2021), pp. 95–115.

46. G. Winter, xia2: An expert system for macromolecular crystallography data reduction. *J. Appl. Cryst.* **43**, 186–190 (2010).

47. A. J. McCoy, R. W. Grosse-Kunstleve, P. D. Adams, M. D. Winn, L. C. Storoni, R. J. Read, Phaser crystallographic software. *J. Appl. Cryst.* **40**, 658–674 (2007).

48. N. Stein, CHAINSAW: A program for mutating pdb files used as templates in molecular replacement. *J. Appl. Cryst.* **41**, 641–643 (2008).

49. P. Emsley, B. Lohkamp, W. G. Scott, K. Cowtan, Features and development of Coot. *Acta Crystallogr. D Biol. Crystallogr.* **66**, 486–501 (2010).

50. D. Liebschner, P. V. Afonine, M. L. Baker, G. Bunkóczi, V. B. Chen, T. I. Croll, B. Hintze, L. W. Hung, S. Jain, A. J. McCoy, N. W. Moriarty, R. D. Oeffner, B. K. Poon, M. G. Prisant, R. J. Read, J. S. Richardson, D. C. Richardson, M. D. Sammito, O. V. Sobolev, D. H. Stockwell, T. C. Terwilliger, A. G.

Urzhumtsev, L. L. Videau, C. J. Williams, P. D. Adams, Macromolecular structure determination using X-rays, neutrons and electrons: Recent developments in Phenix. *Acta Crystallogr. D Struct. Biol.* **75**, 861–877 (2019).

51. C. J. Williams, J. J. Headd, N. W. Moriarty, M. G. Prisant, L. L. Videau, L. N. Deis, V. Verma, D. A. Keedy, B. J. Hintze, V. B. Chen, S. Jain, S. M. Lewis, W. B. Arendall III, J. Snoeyink, P. D. Adams, S. C. Lovell, J. S. Richardson, D. C. Richardson, MolProbity: More and better reference data for improved all-atom structure validation. *Protein Sci.* **27**, 293–315 (2018).

52. Y. Han, X. Fan, H. Wang, F. Zhao, C. G. Tully, J. Kong, N. Yao, N. Yan, High-yield monolayer graphene grids for near-atomic resolution cryoelectron microscopy. *Proc. Natl. Acad. Sci. U.S.A.* **117**, 1009–1014 (2020).

53. K. Naydenova, M. J. Peet, C. J. Russo, Multifunctional graphene supports for electron cryomicroscopy. *Proc. Natl. Acad. Sci. U.S.A.* **116**, 11718–11724 (2019).

54. A. Punjani, J. L. Rubinstein, D. J. Fleet, M. A. Brubaker, cryoSPARC: Algorithms for rapid unsupervised cryo-EM structure determination. *Nat. Methods* **14**, 290–296 (2017).

55. T. Bepler, A. Morin, M. Rapp, J. Brasch, L. Shapiro, A. J. Noble, B. Berger, Positive-unlabeled convolutional neural networks for particle picking in cryo-electron micrographs. *Nat. Methods* **16**, 1153–1160 (2019).

56. M. D. Winn, C. C. Ballard, K. D. Cowtan, E. J. Dodson, P. Emsley, P. R. Evans, R. M. Keegan, E. B. Krissinel, A. G. W. Leslie, A. McCoy, S. J. McNicholas, G. N. Murshudov, N. S. Pannu, E. A. Potterton, H. R. Powell, R. J. Read, A. Vagin, K. S. Wilson, Overview of the CCP4 suite and current developments. *Acta Crystallogr. D Biol. Crystallogr.* **67**, 235–242 (2011).

57. F. Long, R. A. Nicholls, P. Emsley, S. Gražulis, A. Merkys, A. Vaitkus, G. N. Murshudov, AceDRG: A stereochemical description generator for ligands. *Acta Crystallogr. D Struct. Biol.* **73**, 112–122 (2017).

58. M. Schapira, M. Totrov, R. Abagyan, Prediction of the binding energy for small molecules, peptides and proteins. *J. Mol. Recognit.* **12**, 177–190 (1999).

59. E. G. Bligh, W. J. Dyer, A rapid method of total lipid extraction and purification. *Can. J. Biochem. Physiol.* **37**, 911–917 (1959).

60. Y. Z. Tan, P. R. Baldwin, J. H. Davis, J. R. Williamson, C. S. Potter, B. Carragher, D. Lyumkis, Addressing preferred specimen orientation in single-particle cryo-EM through tilting. *Nat. Methods* **14**, 793–796 (2017).
